# SUMO-Specific Protease 2 Is Essential for Modulating p53-Mdm2 in Development of Trophoblast Stem Cell Niches and Lineages

**DOI:** 10.1371/journal.pbio.0060310

**Published:** 2008-12-16

**Authors:** Shang-Yi Chiu, Naoya Asai, Frank Costantini, Wei Hsu

**Affiliations:** 1 Department of Biomedical Genetics, Center for Oral Biology, James P Wilmot Cancer Center, University of Rochester Medical Center, Rochester, New York, United States of America; 2 Department of Pathology, Nagoya University, Nagoya, Japan; 3 Department of Genetics and Development, Columbia University Medical Center, New York, New York, United States of America; Baylor College of Medicine, United States of America

## Abstract

SUMO-specific protease 2 (SENP2) modifies proteins by removing SUMO from its substrates. Although SUMO-specific proteases are known to reverse sumoylation in many defined systems, their importance in mammalian development and pathogenesis remains largely elusive. Here we report that SENP2 is highly expressed in trophoblast cells that are required for placentation. Targeted disruption of SENP2 in mice reveals its essential role in development of all three trophoblast layers. The mutation causes a deficiency in cell cycle progression. SENP2 has a specific role in the G–S transition, which is required for mitotic and endoreduplication cell cycles in trophoblast proliferation and differentiation, respectively. SENP2 ablation disturbs the p53–Mdm2 pathway, affecting the expansion of trophoblast progenitors and their maturation. Reintroducing SENP2 into the mutants can reduce the sumoylation of Mdm2, diminish the p53 level and promote trophoblast development. Furthermore, downregulation of p53 alleviates the SENP2-null phenotypes and stimulation of p53 causes abnormalities in trophoblast proliferation and differentiation, resembling those of the *SENP2* mutants. Our data reveal a key genetic pathway, SENP2–Mdm2–p53, underlying trophoblast lineage development, suggesting its pivotal role in cell cycle progression of mitosis and endoreduplication.

## Author Summary

Genome replication is essential for both expansion of stem cell numbers through mitosis and their maturation into certain specialized cell types through endoreduplication, a unique mechanism for multiplying chromosomes without dividing the cell. An important function of p53 as a guardian of the genome ensures that the genetic information is properly propagated during these processes. In this study, we discovered that mice with disruption of SENP2, an enzyme that removes small molecular signals (called SUMO) that modify a protein's behavior and stability, are unable to form a healthy placenta as a result of deficiencies in the formation of various trophoblast cell types that give rise to the placenta. In the mutants, SUMO modification of Mdm2, a protein that monitors the cellular levels of p53, is deregulated. The loss of SENP2 causes dislocation of Mdm2, leading to aberrant stimulation of p53. The precursor cells known as trophoblast stem cells rely on p53 to proliferate and differentiate into specialized polyploid cells, which contain multiple copies of chromosomes. In *SENP2* mutants, all three trophoblast layers were substantially defective, with the layer containing mainly the polyploid cells most severely affected and diminished. This study reveals a key genetic pathway, SENP2–Mdm2–p53, which is pivotal for the genome replication underlying trophoblast cell proliferation and differentiation.

## Abstract 3

Targeted disruption of SUMO-specific protease 2 in mice reveals that SUMO modulation of the p53/Mdm2 pathway is pivotal for G/S transition of mitosis and endoreduplication during trophoblast development.

## Introduction

The first two distinct lineages to form in the mammalian embryos are the outer trophectoderm and the inner cell mass (ICM) of the blastocyst [1]. The trophectoderm initiates implantation and invasion of the uterus, processes that are essential for placental development [2]. This process depends on the differentiation of trophoblasts, the main and most important cell types in the placenta [3,4]. The trophoblast stem (TS) cells in the mural trophectoderm, distal to the ICM, stop dividing but continue to duplicate their genomes, a mechanism known as endoreduplication. The polyploid trophoblast giant cells (TGCs) then develop and eventually surround the entire fetus [5]. As development proceeds, the trophoblast progenitors give rise to three distinct layers in rodents—labyrinth, spongiotrophoblast and TGCs—to form a functional placenta acting as the maternal–fetal interface [6]. The fetal–placental blood vessels grow in from the allantois to generate the fetal parts of the placental vasculature where the chorioallantoic fusion has occurred [7]. The labyrinth is formed by extensive branching morphogenesis of the labyrinth trophoblast and endothelial cells [8]. The maternal blood passes through the small spaces of the labyrinth, directly contacting the fetal trophoblast cells to ensure exchange between the two blood systems. The labyrinth layer is supported structurally by the spongiotrophoblast cells, which are mainly derived from the ectoplacental cone and which form a layer separating the labyrinth from the TGC. The simplicity of placental cell lineages makes the placenta a valuable model system for understanding general aspects of development, including branching morphogenesis, lineage-specific determination, cell invasion, and polyploidy, crucial for cancer development and metastasis.

SENP2 belongs to a family of proteases that remove a small ubiquitin-related modifier (SUMO) from protein substrates. SUMO (also known as sentrin), which regulates posttranslational modification of proteins, is a member of the ubiquitin-like modifier family [9]. This covalent conjugation process is reversible and highly evolutionary conserved from yeasts to humans [10]. Unlike ubiquitination, which has a well-established role in targeting protein degradation, SUMO modification is involved in protein trafficking, cell cycle, cell survival, and cell death [11]. SUMO conjugation of proteins can alter their function, activity, or subcellular localization. Many sumoylated proteins have been shown to accumulate preferentially in specific complexes such as the nuclear pore and PML (promyelocytic leukemia) bodies [12]. Similar to ubiquitination, sumoylation requires processing, conjugation, and transfer. The transfer process, which covalently conjugates SUMO polypeptides to their targets, is catalyzed by E3 ligases [13]. The reverse desumoylation process is mediated by SUMO proteases. The hallmark of these proteases is the highly conserved carboxyl-terminal SENP domain of ∼200 amino acids. SENP2, which is found in three different alternatively spliced forms, has been localized to the nucleus, cytoplasmic vesicles and PML nuclear bodies [14–16]. Although SENPs have been shown to catalyze SUMO modification in various physiological systems, their roles in mammalian development and pathogenesis are mostly unknown.

We previously discovered an interaction of SENP2 with Axin [17,18], a key signaling regulator for the canonical Wnt pathway. To determine the role of SENP2 in cellular signaling and the importance of SUMO modification in trophoblast development, we initiated a genetic analysis in mice. A SENP2-null mouse strain was created by gene targeting in embryonic stem (ES) cells. We found that the disruption of SENP2 leads to developmental defects in all three trophoblast layers. SENP2 is essential for the G–S transition of both the mitotic and the endoreduplication cell cycles, which control the expansion of trophoblast precursors and the maturation of TGCs, respectively. In the mutants, the loss of SENP2 caused a deregulation of Mdm2, resulting in p53 stimulation. We also present evidence to support an essential role of SENP2 in modulating the p53–Mdm2 circuit that underlies genome replication in mitosis and polyploidy during trophoblast development.

## Results

### Expression of SENP2 in Trophoblast Development

**Figure 1:**
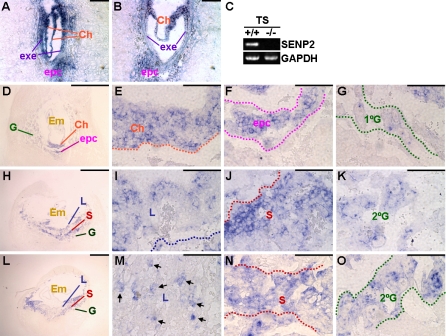
SENP2 Is Expressed in Trophoblast Lineage Development (A and B) In situ hybridization reveals that *SENP2* is expressed in the trophoblast stem cell niches, including extraembryonic ectoderm (exe) , chorion (Ch) and ectoplacental cone (epc) at E7.0 (A) and E7.5 (B). (C) RT-PCR analysis detected the *SENP2* transcript in wild-type (+/+), but not knockout (–/–) TS cells. (D–O) Sections of the E8.5 (D–G), E9.5 (H–K) and E10.5 (L–O) placentas were analyzed by in situ hybridization for the expression of *SENP2*. Expression was detected in major extraembryonic tissues. Low magnification images display the overall expression pattern in developing placentas (D,H,L). High magnification images show expression in specific cell types and layers (E–G,I–K,M–O). The chorion, ectoplacental cone, labyrinth (L), spongiotrophoblast (S), and TGC (G; 1°, primary; 2°, secondary) layers are defined by orange, pink, blue, red, and green broken lines, respectively. Arrows indicate specific expression in mononuclear trophoblasts (cytotrophoblasts) of the labyrinth layer (M). Em, embryo. Scale bars, 1 mm (D,H,L); 100 μm (A,B,E–G,I–K,M–O).

To determine the role of SENP2 and the importance of SUMO modification in trophoblast development, we first examined its expression pattern. Strong expression of *SENP2* was observed in extraembryonic tissues, including extraembryonic ectoderm, chorion and ectoplacental cone, at embryonic day (E)7 (Figure 1A). In extraembryonic ectoderm, its expression started diminishing by E7.5 (Figure 1B). In addition to these stem cell niche sites, we also detected its transcript in TS cells (Figure 1C). At E8.5, *SENP2* maintained its ubiquitous expression in trophoblast cells located in the chorion and ectoplacental cone (Figure 1D–1F). By E9.5 and E10.5, the *SENP2* transcript was detected in all three trophoblast layers: labyrinth, spongiotrophoblast and TGC (Figure 1H and 1L). *SENP2* was expressed in the labyrinth trophoblast cells, which derive from the extraembryonic ectoderm and chorion, upon chorioallantoic fusion at E9.5 (Figure 1I). In the E10.5 labyrinth layer, its expression was specifically localized to cytotrophoblasts (mononuclear trophoblasts), adjacent to the maternal blood cells (Figure 1M). Syncytiotrophoblasts, as well as endothelial and blood cells, appeared to be negative for the staining. In contrast, we found a uniform expression of *SENP2* in spongiotrophoblasts and TGCs (Figure 1J, 1K, 1N, and 1O), which are derivatives of the ectoplacental cone. TGCs include primary and secondary cells, derived from mural trophectoderm and ectoplacental cone (derivatives of polar trophectoderm), respectively. The *SENP2* transcript was detected in both the primary and secondary TGCs (Figure 1G, 1K, and 1O). These results imply an important function of SENP2 in trophoblast progenitors and their development into all three major layers.

### SENP2 Is Required for Extraembryonic Development

A *SENP2*-null allele was created by the targeted insertion of a lacZ reporter with pgk-neo cassette into exon 2 and the deletion of exons 3 to 5 to inactivate all different forms of the *SENP2* gene product (see Materials and Methods for details). The targeted mouse ES cell clones heterozygous for *SENP2* (Figure S1A), obtained by homologous recombination, were then used to obtain the *SENP2^lacZ^* mouse strain (Figure S1B). Mice carrying the targeted allele were subsequently bred with a Zp3-Cre transgenic strain to remove the pgk-neo cassette (*SENP2*-null allele), as confirmed by PCR genotyping analysis (Figure S1C). RT-PCR analyses further showed that the *SENP2*-null allele does not express the *SENP2* transcript, but instead expresses the inserted *lacZ* gene (Figure S1D).

**Figure 2:**
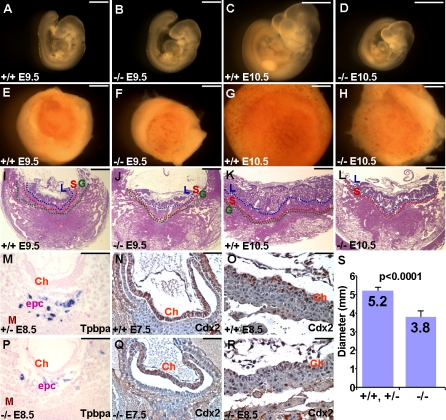
Embryonic and Extraembryonic Abnormalities Caused by SENP2 Deficiency (A–D) Whole mount analysis of the *SENP2^+/+^* (A,C) and *SENP2^–/–^* (B,D) embryos identified growth restriction induced by the deletion of SENP2 at E9.5 (A,B) and E10.5 (C,D). (E–L) The placentas of *SENP2^+/+^* (E,G,I,K) and *SENP2^–/–^* (F,H,J,L) were examined in whole mounts (E–H) or transverse sections (I–L) at E9.5 (E,F,I,J) and E10.5 (G,H,K,L). Labyrinth (L), spongiotrophoblast (S) and TGC (G) layers are defined by blue, red and green broken lines, respectively. Note that TGC layer is missing because of the very few cells present at E10.5 (L). (M–R) Sections of the E7.5–E8.5 extraembryonic tissues were analyzed by in situ hybridization of the ectoplacental cone (epc) marker Tpbpa (M,P) and immunostaining of the chorion (Ch) marker Cdx2 (N,O,Q,R), and counterstaining with nuclear fast red and hematoxylin, respectively. (S) The graph shows the average diameter of the control (+/+, +/–) and mutant (–/–) E10.5 placentas (*p* < 0.0001, *n* = 7). Scale bars, 1 mm (A–H); 500 μm (I–L); 300 μm (M,P); 50; μm (N,O,Q,R).

The *SENP2*-null heterozygous (hereafter referred to as *SENP2^+/–^*) mice were viable and fertile without any noticeable abnormalities. However, we were unable to find *SENP2*-null homozygous (hereafter referred to as *SENP2^–/–^*) newborns, implying that they died prematurely. These results prompted us to investigate whether the loss of SENP2 causes embryonic lethality. The *SENP2^–/–^* embryos appeared to be morphologically indistinguishable from their *SENP2^+/+^* and *SENP2^+/–^* littermates at E9.5 (Figure 2A and 2B). However, the *SENP2^–/–^* embryos were significantly smaller or underdeveloped compared with the *SENP2^+/+^* and *SENP2^+/–^* littermates at E10.5 (Figure 2C and 2D). We could not recover the *SENP2^–/–^* embryos after E11.5. This phenotype is often associated with placental deficiencies, as the embryos begin to rely on maternal supplies upon allantoic fusion at mid gestation. Indeed, the *SENP2^–/–^* placentas were smaller and paler than the controls (Figure 2E–2H). The average diameter of E10.5 placentas reduced from 5.2 mm in controls to 3.8 mm in mutants (Figure 2S, *p* < 0.0001, *n* = 7). Histological analyses revealed a reduction of the TGC layer by E9.5 (Figures 2I and 2J). By E10.5, the thickness of all three trophoblast layers decreased drastically in the SENP2-null mutants (Figures 2K and 2L). The TGC layer, which is the layer most severely affected by the SENP2 mutation, is almost completely missing. The data suggest that SENP2 has a pivotal role in development of all three trophoblast layers.

### Trophoblast Niche Sites Are Defective in SENP2 Nulls

The placental defects caused by SENP2 deficiency suggested that it is critical for trophoblast development. The stem cells derived from the trophectoderm develop into progenitors, which reside in the ectoplacental cone, the extraembryonic ectoderm, and the chorion. We therefore examined whether the *SENP2* deletion interferes with formation of these niche sites. In situ hybridization of Tpbpa, a marker for the ectoplacental cone [19], revealed a drastic reduction of trophoblast progenitors in the mutants (Figure 2M and 2P). The number of trophoblast progenitors, marked by Cdx2 expression [20] was also decreased in the *SENP2^–/–^* chorion and extraembryonic ectoderm (Figure 2N, 2O, 2Q, and 2R). The apparent developmental defects of trophoblast niche sites suggested that SENP2 might have a role in trophoblast stem cell development.

### Effects of SENP2 Deficiency on Labyrinth Layer

**Figure 3:**
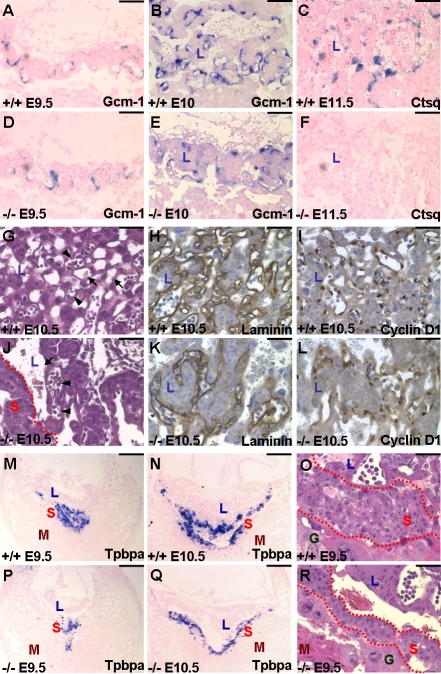
Developmental Defects of the SENP2-Null Labyrinth and Spongiotrophoblast Layers Sections of E9.5–E11.5 placentas were analyzed by in situ hybridization of Gcm1 (A,B,D,E), Ctsq (C,F), or Tpbpa (M,N,P,Q) and counterstained with nuclear fast red (A–F,M,N,P,Q), by histology (G,J,O,R) and immunostaining of laminin (H,K) or cyclin D1 (I,L), and by counterstaining with hematoxylin (H,I,K,L). (A,D) The Gcm-1-positive trophoblast precursors localized to the invasion site were found in both the E9.5 *SENP2^+/+^* and *SENP2^–/–^* placentas. (B,E) At E10, the SENP2 deletion caused an aberrant reduction in the Gcm-1 expressing cells. The Gcm-1-positive syncytiotrophoblasts failed to form an elongated multinuclear structure. (C,F) The Ctsq-positive cytotrophoblasts identified in the E11.5 wild-type labyrinth were missing in the mutant. (G,J) Arrows and arrowheads indicate maternal blood spaces surrounded by trophoblasts and fetal blood spaces surrounded by endothelia, respectively. (H,K) Laminin-labeled basement membrane, highlighting fetal blood spaces. (I,L) Cyclin D1 identified the proliferating endothelial cells. (M,N,P,Q) The number of the Tpbpa-expressing spongiotrophoblasts was drastically reduced by the loss of SENP2. (O,R) The thickness of the spongiotrophoblast layer, defined by broken red lines, decreased significantly. G, TGC; L, labyrinth; M, maternal decidua; S, spongiotrophoblast. Scale bars, 200 μm (A,D); 100 μm (B,C,E,F); 50 μm (G–L,O,R); 500 μm (M,N,P,Q).

A closer examination of the labyrinth layer was performed by analyzing the expression of Gcm1, a labyrinth trophoblast marker that is specifically detected in the chorioallantoic invasion sites and later in the differentiated syncytiotrophoblasts [21]. No obvious difference between *SENP2^+/+^* and *SENP2^–/–^* was observed at E9.5 (Figure 3A and 3D). Gcm-1-positive trophoblast progenitors were clearly identified at the invasion sites. Therefore, fetal vascular invasion was not affected by the deletion. However, deficiencies in labyrinth development of *SENP2^–/–^* embryos were evident at E10. The syncytiotrophoblasts positive for Gcm-1 exhibited punctated staining in the mutant instead of the continuous thin layers seen in the wild type, suggesting that their differentiation is defective (Figure 3B and 3E). By E10.5, the number of the Gcm1-expressing cells was dramatically reduced in the mutants (data not shown). At this stage, *SENP2* expression is restricted to the cytotrophoblasts (Figure 1M), a subtype of TGCs [22]. Therefore, we examined whether cytotrophoblast development was affected by analyzing a cytotrophoblast marker, Ctsq [23]. Indeed, the Ctsq-positive cytotrophoblasts identified in the wild-type labyrinth were completely missing in the mutants (Figure 3C and 3F). Histological analysis further showed that both maternal and fetal blood spaces were enlarged without formation of capillary structures in the SENP2 mutants (Figure 3G and 3J). Immunostaining of laminin [24], a basement membrane protein expressed by endothelial cells that highlight fetal blood spaces, further revealed a failure of branching morphogenesis in the SENP2-null fetal vasculature (Figure 3H and 3K). This might be attributed to a deficiency in endothelial proliferation as the number of cyclin D1-positive cells (proliferation marker only detected in endothelial cells) was decreased in the mutants (Figure 3I and 3L). These data demonstrated that SENP2 is essential for labyrinth trophoblast development in establishment of the maternal and fetal blood spaces. The presence of SENP2 in early trophoblast precursors might regulate the differentiation of specialized cell types at later stages. Alternatively, its function in the cytotrophoblasts could be crucial for proper development of syncytiotrophoblasts and endothelial cells. SENP2 is necessary for development of the labyrinth layer during placentation.

### Spongiotrophoblast Development Is Defective in the Absence of SENP2

We next examined the spongiotrophoblast layer that is affected by the SENP2 deletion. In situ hybridization analysis of Tpbpa [19], a marker for the spongiotrophoblast, revealed that its expressing cells diminished significantly in the mutants at E9.5–E10.5 (Figure 3M, 3N, 3P, and 3Q). Histology confirmed that a rapid expansion of this layer, found in the wild-type placenta, did not occur in the mutants (Figure 3O and 3R). As a result, the SENP2-null spongiotrophoblast layer decreased significantly in volume. Based on the expression of *SENP2* in spongiotrophoblasts (Figure 1J and 1N) and earlier in their precursors at the ectoplacental cone (Figure 1A, 1B, and 1F), it is most likely that the abnormalities are primarily due to its deletion in these tissues. Therefore, spongiotrophoblast development requires SENP2 and its disruption induces abnormalities in the spongiotrophoblast layer.

### Impaired Development of TGC in the SENP2-Null Placentas

**Figure 4:**
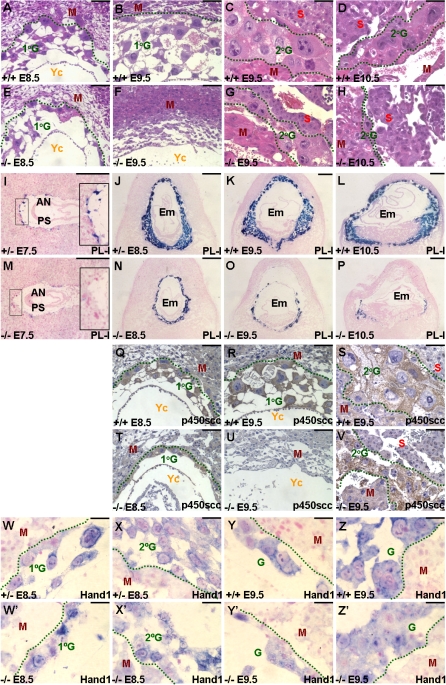
Development of TGCs is Impaired in the SENP2 Mutants (A–H) Histological analysis of the *SENP2^+/+^* (A–D) and *SENP2^–/–^* (E–H) placentas revealed impaired development of both primary (1°G; A,B,E,F) and secondary (2°G; C,D,G,H) caused by SENP2 ablation at E8.5 (A,E), E9.5 (B,C,F,G) and E10.5 (D,H). (I–P,W–Z′) TGC development was examined by in situ hybridization analysis of specific markers PL-I (I–P) and Hand1 (W–Z and W′–Z′) at the stages shown (E7.5–E10.5). Stained (blue) sections were counterstained with nuclear fast red. In (I, M), enlargements of the left insets are shown on the right insets. (Q–V) Immunostaining of p450scc characterized the TGC in *SENP2^+/+^* (Q–S) and *SENP2^–/–^* (T–V) at E8.5 (Q,T), and E9.5 (R,S,U,V). Immunostained (brown) sections were counterstained (blue) with hematoxylin. The TGC layers are defined by broken green lines (A–E,G,H,Q–Z,W′–Z′). AN, anterior neural fold; Em, embryo; G, TGC layer; M, maternal decidua; PS, primitive streak; S, spongiotrophoblast layer; Yc, yolk sac cavity. Scale bars, 500 μm (I–P); 100 μm (A,B,E,F,Q,R,T,U); 50 μm (C,D,G,H,S,V,W–Z,W′–Z′).

Consistent with its expression in early trophoblast development, histological analyses revealed a severe abnormality in the TGC layer (Figure 4A–4H). The SENP2-null primary TGCs were reduced at E8.5 and completely missing at E9.5 (Figure 4A, 4B, 4E, and 4F). Similarly, the number of secondary TGCs was decreased at E9.5 and almost disappeared at E10.5 (Figure 4C, 4D, 4G, and 4H). In addition, the size of TGCs was significantly smaller in the SENP2 mutants (Figure 4D and 4H). The analyses of TGC markers [19,25], including PL-I (Figure 4I–4P), PL-II (unpublished data), and p450scc (Figure 4Q–4V), confirmed that the TGC cell numbers were dramatically decreased in the SENP2 mutants at all stages examined. We next examined the initiation of TGC differentiation by in situ hybridization of Hand1. Hand1 is required for cell fate determination of TGC, as mice without Hand1 lack TGCs [26]. Hand1 expression was detected in the SENP2-null TGCs, suggesting that the initial induction of TGCs was not affected by the loss of SENP2 (Figure 4W–4Z and 4W′–4Z′). However, later developmental processes of TGC were impaired in the mutants.

**Figure 5:**
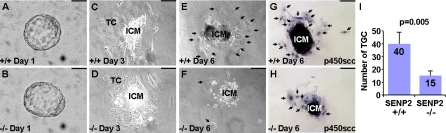
In Vitro Differentiation of SENP2-Null Blastocysts into Trophoblast Cells Is Defective (A–H) Isolated *SENP2^+/+^* (A) and *SENP2^–/–^* (B) blastocysts were cultured for trophoblast differentiation in vitro. Images were taken at culturing day 1 (A,B), day 3 (C,D) and day 6 (E–H). TS cells, outgrowing from the trophectoderm, differentiated into a single trophoblast cell (TC) layer, whereas the ICM formed aggregates and sat on top of the trophoblast cells (C,D). Arrows indicated TGCs present in the cultures (E,F). The cultures were then analyzed by immunostaining of a trophoblast specific marker p450scc (brown) and counterstaining of hematoxylin (blue) on day 6 (G,H). (I) The graph represents the average number of TGC present in the *SENP2^+/+^* and *SENP2^–/–^* cultures (*p* = 0.005, *n* = 6). Scale bars, 200 μm (E–H); 100 μm (C,D); 50 μm (A,B).

The abnormal development of TGC caused by SENP2 deficiency was further tested using an in vitro differentiation analysis. The *SENP2^+/+^* and *SENP2^–/–^* blastocysts were isolated at E3.5, and cultured to induce TGC differentiation. TS cells growing out from the trophectoderm soon attached to the cultured plates, differentiated, and formed a single trophoblast layer. No noticeable difference was observed between the *SENP2^+/+^* and *SENP2^–/–^* blastocysts before hatching (Figure 5A and 5B). About equal amounts of ICM and trophoblast cells developed after 3 d in culture (Figure 5C and 5D). However, although the differentiated TGCs were evident in the *SENP2^+/+^* cultures, their number was significantly reduced in the *SENP2^–/–^* cultures after 6 d (Figure 5E–5H). The average number of TGC dropped from 40 in the *SENP2^+/+^* culture to 15 in the *SENP2^–/–^* (Figure 5I, *p* = 0.005, *n* = 6). Consistent with our in vivo findings, these data suggest that TGC differentiation is severely affected by the loss of SENP2. The results suggest an essential role for SENP2 in TGC development during early placentation.

### Cell Cycle Defects Caused By SENP2 Deficiency

**Figure 6:**
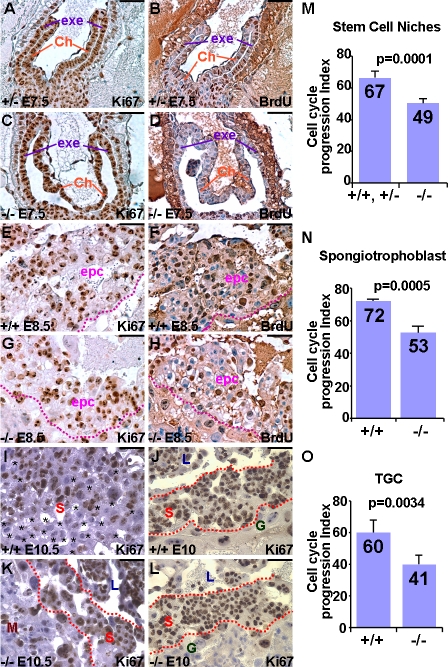
Defects in Trophoblast Cell Cycle Progression Caused by SENP2 Deficiency (A–H) In sections of the E7.5 and E8.5 *SENP2^+/+^*, *SENP2^+/–^* and *SENP2^–/–^* extraembryonic structures, Ki67 staining identified trophoblast progenitors undergoing cell cycle progression at the trophoblast stem cell niches (A,C,E,G). BrdU labeling for 1 h, performed on adjacent sections, detected the progression rate at S phase (B,D,F,H). (I–L) *SENP2^+/+^* (I,J) and *SENP2^–/–^* (K,L) spongiotrophoblasts were analyzed by immunostaining of Ki67 at E10.5 (I,K) and E10 (J,L). Asterisks indicated the Ki67-negative cells in the *SENP2^+/+^* spongiotrophoblast layer (I). The adjacent section of E10 placentas were stained with anti-BrdU to obtain the progression rate. (M) The graph represents cell cycle progression index, which is the average progression rate among actively cycling cells (% of BrdU divided by % of Ki67 × 10^2^), at all stem cell niches, extraembryonic ectoderm, chorion and ectoplacental cone (*p* = 0.0001, *n* = 6). The positive and negative cells were counted to obtain the percentages of BrdU- and Ki67-positive cells. (N,O) The graphs represent the cell cycle progression index of the spongiotrophoblast (N; *p* = 0.0005, *n* = 3) and TGC (O; *p* = 0.0034, *n* = 4) layers. Ch, chorion; epc, ectoplacental cone; exe, extraembryonic ectoderm; G, TGC; M, maternal decidua; L, labyrinth; S, spongiotrophoblast. Scale bars, 50 μm (A–L).

The SENP2 mutation led to abnormalities in trophoblast progenitors at niche sites and their development into all three major trophoblast lineages. These findings imply that SENP2 might have a general role in cellular regulations important for expansion of precursors and their differentiation. We speculated that decreases in the numbers of trophoblast progenitors and specialized cell types might be due to alterations in cell survival. However, we failed to detect differences in apoptosis caused by the mutation in trophoblast stem cell niches and all three major trophoblast layers in vivo, or in TS cell culture in vitro (Figure S2). We then examined whether SENP2 has an important function in the cell cycle. Investigating the expansion of trophoblast progenitors at the niches revealed a deficiency in their cell cycle progression. The expression of Ki67, a marker detected in all phases of mitotic cells [18], was detected in virtually all trophoblast progenitors in stem cell niches, including extraembryonic ectoderm, chorion, and ectoplacental cone (Figure 6A, 6C, 6E, and 6G), suggesting that they are actively cycling cells. We next examined the cell cycle progression rate among actively cycling cells by measuring the DNA synthesis rate at S phase using BrdU labeling [18] for 1 h (Figure 6B, 6D, 6F, and 6H). BrdU incorporation specifically measures the rate of cell cycle progression at S phase, whereas Ki67 identifies all phases of mitotic cells. The cell cycle progression index (% BrdU-positive cells / % of Ki67-positive cells × 10^2^) among actively cycling cells decreased 18 units in the mutants (*SENP2^+/+^* and *SENP2^+/–^*, 67; *SENP2^–/–^*, 49; *p* = 0.0001, *n* = 6) in the stem cell niches (Figure 6M). These data suggest a delay in cell cycle progression of trophoblast progenitors caused by the SENP2 deletion.

Next, we determined whether similar deficiencies also affect development of the spongiotrophoblast layer. We found that this layer expanded rapidly in the wild-type placenta, but not in the mutants, between E9.5 and E10.5 (data not shown). A portion of the *SENP2^+/+^* spongiotrophoblasts exited the cell cycle at E10.5 (Figure 6I), whereas almost all of the *SENP2^–/–^* spongiotrophoblasts remained Ki67-positive (Figure 6K). In E10 *SENP2^+/+^* and *SENP2^–/–^* placentas, spongiotrophoblasts were all positive for Ki67, indicating that they are actively cycling cells (Figure 6J and 6L). However, the cell cycle progression index, which mainly reflects the BrdU incorporation rate, was reduced from 72 in the controls to 53 in the SENP2 mutants (*p* = 0.0005, *n* = 3) (Figure 6N). To examine whether cycling of TGC was also affected by SENP2 deficiency, we determined its cell cycle progression index (Figure 6O). The cell cycle progression index of TGC decreased from 60 in *SENP2^+/+^* to 41 in *SENP2^–/–^* (*p* = 0.0034, *n* = 4). Taken together, these results suggest that cell cycle progression was defective in all stem cell niches, and the spongiotrophoblast and TGC layers, of *SENP2* mutants. The SENP2 mutant cells were trapped or arrested in the cell cycle.

### SENP2 Is Essential for the G–S Transition in Mitosis and Endoreduplication

**Figure 7:**
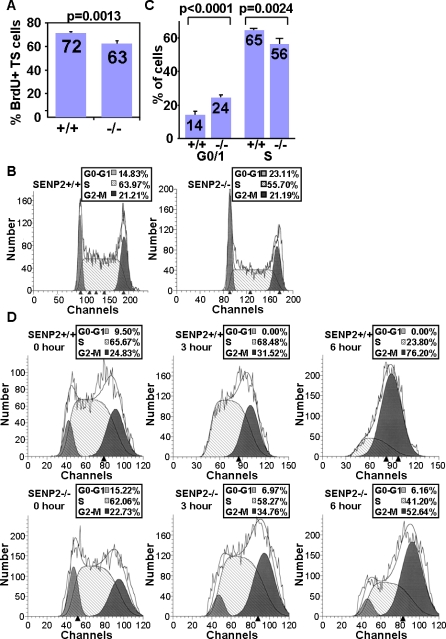
SENP2 Is Critical for the G1–S Transition of Mitotic Division in TS Cells (A) BrdU labeling for 1 h measured the proliferation rate of the *SENP2^+/+^* and *SENP2^–/–^* TS cells in vitro. The graph shows the average percentages of the BrdU-positive cells in three independent experiments (*p* = 0.0013, *n* = 3). (B,C) Flow cytometric analysis of the PI-stained *SENP2^+/+^* and *SENP2^–/–^* TS cells to determine their cell cycle profiles. The result shown in (B) is a representative of four independent experiments, and the graph in (C) shows the average percentage of the G0–G1 and S populations (*n* = 4). A consistent increase in the G0–G1 population (*p* < 0.0001) and decrease in the S population (*p* = 0.0024) was detected in the SENP2 mutants. (D) The *SENP2^+/+^* and *SENP2^–/–^* TS cells were treated with nocodazole for 0, 3 and 6 h as indicated. Flow cytometric analyses showed that there was a delay in synchronizing the *SENP2^–/–^* cells upon the nocodazole treatment.

To further examine stem cell expansion and development, we derived a number of *SENP2^–/–^* TS cell lines from blastocysts. Immunostaining analyses of Oct4 (an ES cell marker) [27] and Cdx2 (a TS cell marker) [20] confirmed that we were able to successfully establish the SENP2-null TS cell lines (Figure S3). The proliferation rate (BrdU labeling for 1 h) of the SENP2-null TS cells in vitro was also reduced, compared to that of the wild-type cells (*p* = 0.013, *n* = 3) (Figure 7A). Although the deficiency in cell cycle progression was also demonstrated using the TS cells in vitro, the degree of severity was reduced compared with that seen in the in vivo studies. As we were aware, the in vitro system does not always recapitulate the dynamic developmental processes that occur in vivo. Nevertheless, because of the limited materials available from the early stages of placenta, the TS cell culture does provide a valuable system to further those of our investigations that are otherwise impossible to perform in vivo.

To investigate whether a specific phase of the cell cycle was defective, we then determined the cell cycle profiles of the *SENP2^+/+^* and *SENP2^–/–^* TS cells by flow cytometry analysis of PI (propidium iodide) stained cells. There was no significant difference in the cell population of G2–M between *SENP2^+/+^* and *SENP2^–/–^* cells (Figure 7B). However, in the SENP2 nulls, the percentage of cells in G0–G1 was increased (*p* < 0.0001, *n* = 4) but the percentage in S was decreased (*p* = 0.0024, *n* = 4) (Figure 7B and 7C). This implied that the mutant cells were affected at the G1–S transition. To test this hypothesis, we used nocodazole, a microtubule depolymerizing agent, to block cell division at M phase. Nocodazole was effective in synchronizing the *SENP2^+/+^* TS cells at G2–M after 6 h (Figure 7D). However, if cells were arrested or trapped in the G1–S phase and unable to pass through the cell cycle, there would be a delay in synchronizing cells by the nocodazole treatment. Indeed, there were still ∼7% of the G0–G1 cells in *SENP2^–/–^*, but none in *SENP2^+/+^*, 3 h after the treatment. After the 6 h treatment, a significant number (6.16%) of the *SENP2^–/–^* TS cells remained in G0–G1 (Figure 7D). Even after 24 h, this population arrested in G0–G1 was still present (data not shown). The results suggest that SENP2 has a pivotal role in TS cell cycle progression and the G1–S checkpoint might be affected by the SENP2 ablation.

**Figure 8:**
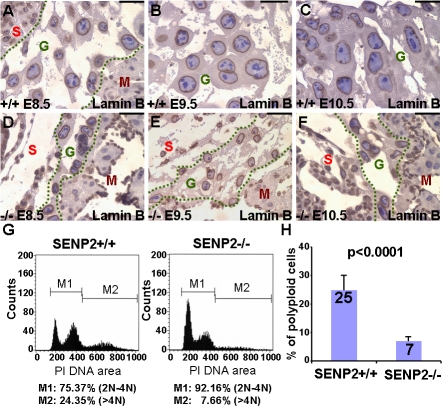
SENP2 Is Required for Trophoblast Maturation (A–F) Immunostaining of the E8.5 (A,D), E9.5 (B,E) and E10.5 (C,F) *SENP2^+/+^* (A–C) and *SENP2^–/–^* (D–F) placentas with lamin B, which marks nuclear envelopes, shows the size of nuclei. The TGC layers are defined by broken green lines. The stained (brown) sections were counterstained (blue) with hematoxylin. Note that the SENP2-null TGCs (D–F) contain smaller nuclei with less dotted staining (representing nucleoli and heterochromatin) than the controls (A–C). (G) Endoreduplication is impaired by the loss of SENP2. The *SENP2^+/+^* and *SENP2^–/–^* TS cells were induced for differentiation into TGCs in vitro. Flow cytometric analysis of the differentiated *SENP2^+/+^* and *SENP2^–/–^* cells, stained with PI, was used to measure their DNA contents (M1, two to four copies; M2, more than four copies). The diagram in (G) is a representative of five independent experiments; the average percentages of the *SENP2^+/+^* and *SENP2^–/–^* polyploid cells in all five cultures is presented in (H) (*p* < 0.0001, *n* = 5). G,TGC layer; M, maternal decidua; S, spongiotrophoblast layer. Scale bars, 50 μm.

Immunostaining of nuclear envelopes with lamin B [28] revealed that nuclei of the *SENP2^–/–^* TGCs were significantly smaller (Figure 8A–8F). In addition, the mutant TGC nuclei contained smaller and fewer blue dots upon hematoxylin staining (Figure 8A–8F), suggesting that the DNA content might be reduced. These abnormalities are likely caused by a deficiency in endopolyploidy. An important specialized process for TGC maturation is endoreduplication, whereby the genome is amplified without a complete mitosis. The endoreduplication cycle requires only the G and S phases [29]. To examine the possibility of a defect in endoreduplication, we induced the TS cells to undergo TGC differentiation in vitro by removal of FGF4, heparin, and mouse embryonic fibroblast (MEF)-conditioned medium (see Materials and Methods and [30]). Flow cytometric analysis of the differentiated cells stained with PI showed that the percentage of cells with higher DNA contents (>4N) was drastically reduced in the SENP2 mutants (Figure 8G). The average percentage of polyploid cells reduced from 25% (*SENP2^+/+^*) to 7% (*SENP2^–/–^*) (*p* < 0.0001, *n* = 5) (Figure 8H). Therefore, the loss of SENP2 induced a severe deficiency in endopolyploidy. SENP2 apparently has a dual role in regulating the G–S transition of mitotic division and endoreduplication during TS cell proliferation and differentiation, respectively.

### Disruption of SENP2 Alters the p53–Mdm2 Circuit in Trophoblast Development

**Figure 9:**
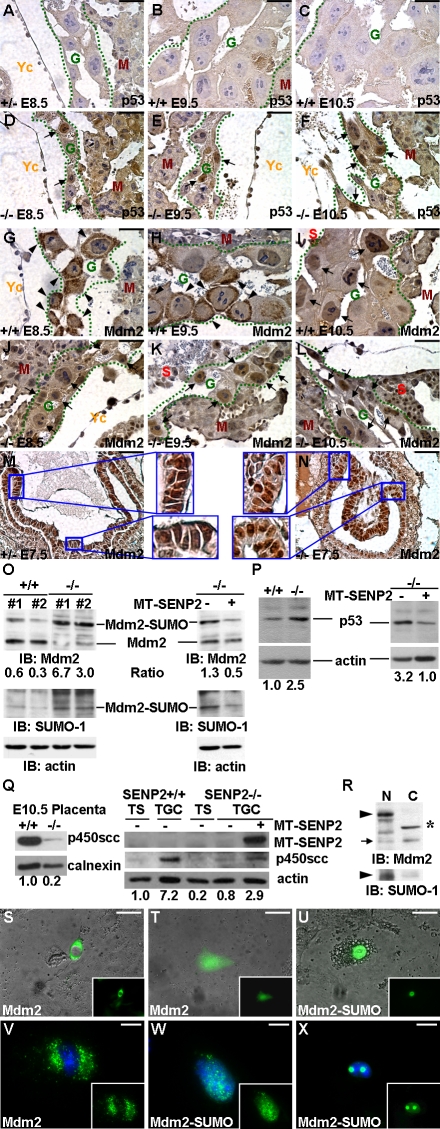
SENP2 Regulates the p53–Mdm2 Circuit During Trophoblast Development (A–N) Sections of the E7.5 (M,N), E8.5 (A,D,G,J), E9.5 (B,E,H,K) and E10.5 (C,F,I,L) *SENP2^+/+^* or *SENP2^+/–^* (A–C,G–I,M) and *SENP2^–/–^* (D–F,J–L,N) placentas were stained with an anti-p53 (A–F) or anti-Mdm2 antibody (G–N). The stained (brown) sections were counterstained with hematoxylin (blue). (D–F) Nuclear accumulations of p53 (arrows) were detected in the SENP2 mutants. (G–I) In the *SENP2^+/+^* TGCs, Mdm2 predominantly accumulated in the cytoplasm at E8.5 and E9.5 (arrowheads; G,H), but in the nucleus at E10.5 (arrows; I). (J–L) Nuclear accumulations of Mdm2 were found throughout the *SENP2^–/–^* TGC development at E8.5–E10.5 (arrows; J,K,L). The TGC layers are defined by broken green lines. (M,N) Mdm2 showed clear nuclear localizations in the *SENP2^–/–^* trophoblast progenitors at the niche sites (N), whereas it was evenly distributed in the controls (M). Enlargements of the insets are shown. (O) SUMO modification of Mdm2 is regulated by SENP2. Immunoblot analysis with anti-Mdm2 and anti-SUMO-1 antibodies shows that Mdm2 accumulated in its sumoylated state (Mdm2–SUMO) in the *SENP2^–/–^* trophoblast cells. Two different cell lines (#1 and #2) were examined. The Mdm2–SUMO band could also be detected by immunoprecipitation–immunoblot with anti-Mdm2 and anti-SUMO-1 antibodies (data not shown). Reintroduction of SENP2 into the *SENP2^–/–^* TS cells diminished the Mdm2–SUMO level. Actin level also was analyzed as a loading control. The number indicates the ratio of Mdm2–SUMO and Mdm2. (P) The p53 protein level is regulated by SENP2. Protein lysates were isolated from the *SENP2^+/+^* and *SENP2^–/–^* TS cells with or without transfection of MT–SENP2. Immunoblot analysis with an anti-p53 antibody revealed the steady state levels of p53 and actin (loading control). Inactivation of SENP2 induced an accumulation of p53 in trophoblasts. Reintroduction of SENP2 down regulated p53 in the SENP2-null mutants. The number represents the expression level of p53 in *SENP2^–/–^* relative to that in *SENP2^+/+^*. (Q) SENP2 is necessary and sufficient to induce trophoblast differentiation. Protein lysates were isolated from the *SENP2^+/+^* and *SENP2^–/–^* placentas at E10.5, and the *SENP2^+/+^* and *SENP2^–/–^* TS cells with or without transfection of MT–SENP2. The TS cells were cultured in differentiation media for 6 d to obtain the differentiated TGCs. Immunoblot analysis with an antibody that recognizes either p450scc or MT revealed the steady state protein level. The levels of ER protein calnexin and actin were analyzed as loading controls. The number shows the quantitative difference in p450scc expression. (R) Preferential accumulations of Mdm2 (arrow) and Mdm2–SUMO (arrowheads) in TS cells. Nuclear (N) and cytoplasmic (C) extracts of *SENP2^–/–^* were analyzed by immunoblot with anti-Mdm2 and anti-SUMO-1 antibodies. Asterisk indicates non specific reaction detected after cell fractionation. (S–X) Mdm2 and Mdm2–SUMO are differentially localized in the cell. TS cells transfected by the GFP-tagged Mdm2 (S,T,V) or Mdm2–SUMO-1 fusion (U,W,X) under control of a CMV promoter were analyzed by GFP analysis with either phase contrast (S–U) or by immunofluorescence microscopy (blue, DAPI) (V–X). G, TGC layer; M, maternal decidua; S, spongiotrophoblast layer; Yc, yolk sac cavity. Scale bars, 50 μm (A–N,S–U); 20 μm (V–X).

The cell cycle defects led us to investigate potential downstream targets involved in trophoblast development. We specifically focused on those regulators shown to be conjugated by SUMO. Previous reports showed that SENP2 (also known as Axam) modulates the canonical Wnt pathway by interacting with its signaling molecules [14,31]. Even though this led us to identify SENP2 through its binding to Axin initially, we failed to detect any alteration of Wnt signaling in the SENP2 mutants. Nor were we able to show other alternative pathways critical for placentation, e.g., MAPK and SAPK [32–36], to be involved in the SENP2-dependent developmental processes. However, when p53 was examined by immunostaining, we detected an aberrant accumulation in the nuclei of the developing *SENP2^–/–^* TGCs at E8.5–E10.5 (Figure 9D–9F). In contrast, the *SENP2^+/+^* TGCs showed no detectable, or very low if any, p53 at these stages (Figure 9A–9C). The results implied that there is a deficiency in p53 regulation caused by the SENP2 deletion. Degradation of p53 is mediated by ubiquitination-dependent proteolysis. Mdm2, a RING finger E3 ubiquitin ligase that binds to p53, has an essential role in this process [37–40]. We therefore tested whether the loss of SENP2 had an effect on Mdm2. Immunostaining of Mdm2 revealed its localization in both the *SENP2^+/+^* cytoplasm and the nucleus during early stages (E8.5–E9.5) of TGC development (Figure 9G and 9H). However, Mdm2 was mainly located to the nuclei of the terminally differentiated TGCs at E10.5 (Figure 9I). The differential subcellular distribution of Mdm2 implies that it might be critical for development of TGCs. In contrast, Mdm2 accumulated in nuclei throughout TGC development in the absence of SENP2 (Figure 9J–9L). The prominent cytoplasmic staining was lost in the mutants at E8.5–E9.5 (Figure 9J and 9K). Furthermore, the loss of SENP2 also affected Mdm2 localization in the stem cell niche sites, such as extraembryonic ectoderm and chorion. Mdm2 clearly accumulated in the nuclei of the *SENP2^–/–^* trophoblast progenitors, but was evenly distributed in the whole cells of the controls (Figure 9M and 9N). Similar nuclear accumulations of Mdm2, affecting the p53 level, were also detected in the *SENP2^–/–^* labyrinth and spongiotrophoblast layers (data not shown). Therefore, Mdm2 appeared to be aberrantly localized in the stem cell niches and all three major layers of trophoblast during early embryogenesis. The data suggest that SENP2 is required for proper localization of Mdm2 and degradation of p53. Disturbance of SUMO modification by the SENP2 deletion thus causes deregulation of the p53–Mdm2 pathway, leading to deficiencies in mitotic and endoreduplication cell cycle progression and abnormal trophoblast development.

The accumulation of p53 in the nuclei of SENP2-null placentas implied that SENP2 negatively modulates the p53–Mdm2 circuit. To determine the role of p53–Mdm2 in trophoblast development, we investigated whether SENP2 modulates Mdm2 and p53 at the posttranscriptional level. In addition to altering the subcellular distribution of Mdm2, the loss of SENP2 had an effect on posttranslational modification of Mdm2. The loss of SENP2 disturbed desumoylation of Mdm2. In the *SENP2^–/–^* TS cells, Mdm2 accumulated in the SUMO conjugated state (Figure 9O). The loss of SENP2 disturbed the ratio of Mdm2 and Mdm2–SUMO. The sumoylated Mdm2 could also be detected by an anti-SUMO-1 antibody (Figure 9O) as well as immunoprecipitation–immunoblot analysis using anti-Mdm2 and anti-SUMO-1 antibodies (data not shown). We encountered a technical problem in determining the actual amount of the sumoylated Mdm2 by immunoprecipitation–immunoblot analysis. This is likely because desumoylation occurs rapidly in isolated cell extracts whereas immunoprecipitation requires proteins in a native conformation. Therefore, a straight immunoblot assay appears to be better suited for quantitative measurements. To determine whether SUMO modification of Mdm2 is regulated by SENP2, a plasmid expressing a Myc-tagged SENP2 (MT–SENP2) under the control of a CMV promoter was transiently transfected into the mutants. The reintroduction of SENP2 altered the ratio of Mdm2 and Mdm2–SUMO and diminished the level of Mdm2–SUMO, suggesting that its desumoylation is modulated by SENP2 (Figure 9O). Immunoblot analysis also revealed an elevation of p53 caused by the SENP2 deletion in TS cells (Figure 9P). Although p53 is known to be sumoylated, we did not detect obvious accumulations of the SUMO-conjugated form caused by the SENP2 ablation. We then tested whether SENP2 is required to mediate the downregulation of p53 by overexpression of MT–SENP2. Consistent with our hypothesis, p53 levels were significantly reduced in the SENP2-null cells transiently transfected by MT–SENP2 (Figure 9P).

To further confirm that the loss of SENP2 was the primary cause of the trophoblast defects, we reintroduced MT–SENP2 into *SENP2^–/–^* cells. To determine the differentiation process affected by SENP2 at a more quantitative level, we examined the expression of a TGC marker, p450scc, by immunoblot analysis. The expression of p450scc was drastically reduced in *SENP2^–/–^* placentas, confirming the TGC developmental defects (Figure 9Q). The expression of p450scc was not detectable in *SENP2^+/+^* TS cells but was highly increased in the differentiated TGCs, suggesting the success of the in vitro culture system (Figure 9Q). We did not detect a great induction of p450scc in the differentiated *SENP2^–/–^* cells, consistent with our in vivo findings (Figure 9Q). The reintroduction of MT–SENP2 in the SENP2 mutants led to an induction of p450scc upon TGC differentiation (Figure 9Q). The p450scc induction level did not reach that of the *SENP2^+/+^* TGCs, most likely due to the transfection efficacy, in that not all of the mutants were transfected. Nevertheless, these data demonstrate that reintroducing SENP2 into the *SENP2^–/–^* TS cells can promote their differentiation into TGCs. This suggests that SENP2 inactivation is the cause of the trophoblast developmental defects observed in the mutants. An aberrant stimulation of p53 might be responsible for the SENP2-null defects in mitotic division and polyploidy.

In the SENP2 mutants, the dislocation of Mdm2 implied that its distribution is regulated by the SUMO pathway. We therefore investigated whether Mdm2 localization is affected by SUMO. First, immunoblot analysis after cell fractionation showed that sumoylated Mdm2 is found preferentially in the nuclear fraction of *SENP2^–/–^* cells (Figure 9R). Next, we examined whether SUMO conjugation alters the subcellular distribution of Mdm2 in live cells. GFP analysis of TS cells transiently expressing GFP-tagged Mdm2 or Mdm2–SUMO-1, revealed their preferential localization. We found that Mdm2 mainly accumulated in the cytoplasm (Figure 9S and 9V), with occasional distribution to the whole cell (Figure 9T). However, Mdm2–SUMO-1 displayed a clear nuclear accumulation (Figure 9U), with either a punctated (Figure 9W) or a nucleolar (Figure 9X) staining pattern. Similar results were also obtained by the use of Mdm2–SUMO-1^GG96–97Δ^, a mutant lacking the last two glycine residues of SUMO-1, which prevent further conjugation that might affect subcellular distribution (data not shown). Therefore, the SENP2 mediated SUMO modification of Mdm2 appears to be crucial for its subcellular trafficking.

### The Requirement for p53 in Mediating the Deficiencies of SENP2-Null Mutants

**Figure 10:**
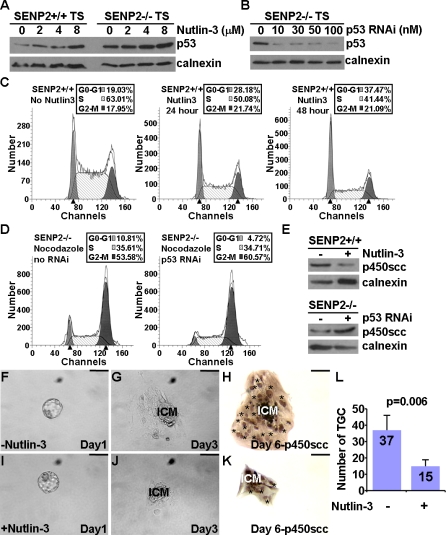
Repression of p53 Is Necessary and Sufficient to Promote Trophoblast Proliferation and Differentiation (A,B) Nutlin-3 stimulates p53 in a dosage-dependent manner (A) and accumulation of p53 in the SENP2-nulls can be knocked down by RNAi (B). Protein lysates, isolated from *SENP2^+/+^* and *SENP2^–/–^* TS cells treated with Nutlin-3 (A) or transfected by p53 RNAi (B), were analyzed for the p53 expression by immunoblot. Calnexin was used as a loading control. (C) Activation of p53 by Nutlin-3 caused a delay in the G1–S transition. Flow cytometric analysis of PI-stained *SENP2^+/+^* TS cells determined the cell cycle profiles without Nutlin-3 or affected by Nutlin-3 treatment for 24 or 48 h. The Nutlin-3 (8 μM) treatment induced a cell cycle arrest at G1–S. (D) The p53 RNAi treatment alleviates the cell cycle defects caused by the SENP2 deletion. The *SENP2^–/–^* TS cells with or without p53 RNAi (100 nM) were treated with nocodazole for 30 h. Flow cytometric analyses revealed that the cell population arrested in G0–G1 of *SENP2^–/–^* TS cells was reduced by the p53 knockdown. (C,D) are representatives of two independent experiments. (E) Stimulation of p53 is necessary and sufficient to inhibit trophoblast maturation. Protein lysates, isolated from *SENP2^+/+^* and *SENP2^–/–^* cells with or without the Nutlin-3 (8 μM) treatment and the transfection of p53 RNAi, were analyzed by immunoblot analysis for the expression of a TGC marker p450scc. Calnexin was used as a loading control. (F–K) Nutlin-3 inhibits differentiation of blastocysts into TGCs. Isolated blastocysts were cultured for trophoblast differentiation in the absence (F–H) and presence (I–K) of Nutlin-3 (8 μM) in vitro. Images were taken at culturing day 1 (F,I), day 3 (G,J) and day 6 (H,K). The cultures were then analyzed by immunostaining of a TGC-specific marker p450scc (brown) and counterstaining by hematoxylin (blue) on day 6 (H,K). Asterisks indicate TGCs. (L) The graph shows the average number of TGC present in the cultures (*p* = 0.006, *n* = 4). Scale bars, 100 μm (F–K).

To address the importance of p53 in mediating the SENP2-null phenotype, we tested whether p53 activation is necessary and sufficient to affect trophoblast proliferation and differentiation. We used both gain-of-function and loss-of-function analyses. Nutlin-3 is a potent small-molecule antagonist of Mdm2, which binds to the p53-binding pocket of Mdm2 and prevents its interaction, thereby stabilizing p53. We first determined that the Nutlin-3 treatment of the *SENP2^+/+^* cells could elevate p53 in a dosage-dependent manner, but, most importantly, to reach the level detected in the *SENP2^–/–^* TS cells (Figure 10A). To examine whether the p53 elevation induced G1–S arrest, TS cells were treated with Nutlin-3. A cell cycle profiling assay showed that the Nutlin-3 treatment caused the wild-type TS cells to accumulate in G0–G1 phase, similar to the *SENP2^–/–^* TS cells (Figure 10C). Next, we examined whether the elevated level of p53 interfered with the differentiation process. In the *SENP2^+/+^* TS cells induced for TGC differentiation, Nutlin-3 significantly reduced the expression of the TGC marker p450scc (Figure 10E), and prevented TGC differentiation (Figure 10F–10K). The average number of TGC decreased significantly in the presence of Nutlin-3 (Figure 10L, *p* = 0.006, *n* = 4). These results support the hypothesis that stimulation of p53 by alteration in Mdm2 activity induces phenotypic defects in trophoblast proliferation and differentiation, resembling those observed in the SENP2 mutants.

To determine whether downregulation of p53 was able to alleviate the trophoblast deficiencies caused by the SENP2 ablation, we knocked down its cellular levels using an RNA interference (RNAi) approach. First, immunoblot analysis showed that the p53 RNAi treatment successfully diminished its levels in the *SENP2^–/–^* TS cells (Figure 10B). The p53 RNAi treatment also promoted the G1–S transition of the *SENP2^–/–^* TS cells arrested in G0–G1 (Figure 10D). Furthermore, downregulation of p53 enhanced TGC differentiation of the *SENP2^–/–^* cells, as determined by the expression of p450scc (Figure 10E). These data demonstrated that stimulation of p53 is not only necessary to mediate the SENP2-null defects, but is also sufficient to induce deficiencies in expansion of trophoblast stem cells and their maturation.

## Discussion

**Figure 11:**
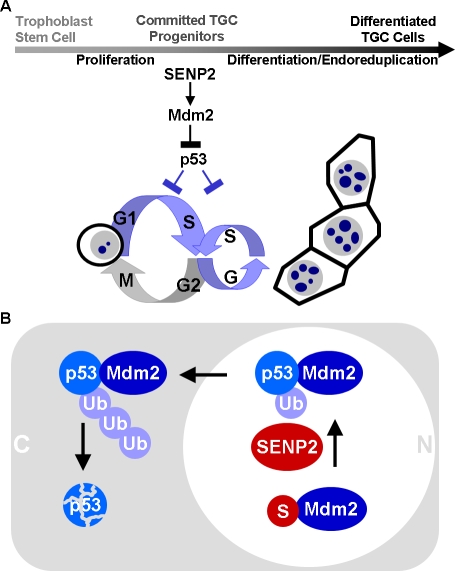
Model for the SENP2–Mdm2–p53 Pathway in Trophoblast Development (A) Diagram illustrating the p53–Mdm2 circuit regulated by SENP2 in the trophoblast cell cycle. Stimulation of Mdm2 by SENP2 leads to degradation of p53. Cellular levels of p53 control the G–S transition that has a dual role in TGC development. The G–S phase is required for both mitotic division (cell cycle: G1, S, G2, and M) and endoreduplication (endocycle: G and S only) during expansion of trophoblast stem cells and maturation of trophoblasts, respectively. Although a low p53 level is essential for stem cell proliferation, inhibition of p53 is required upon differentiation. (B) Schematic representation for the mechanism underlying the regulation of p53 and Mdm2 by the SUMO pathway. SENP2 activates Mdm2 by removing SUMO that permits the modulation of p53 by Mdm2 in the nucleus. The ubiquitin-conjugated p53 is then degraded in the cytoplasm.

This study demonstrates an essential role of SENP2 in trophoblast lineage development during placentation. All three major trophoblast layers were affected by SENP2 deficiency. Our data provide an important connection between SENP2 and the p53–Mdm2 pathway in trophoblast development. The loss of SENP2 caused a deficiency in the G–S transition, which is required for both the mitotic cell cycle (containing G1, S, G2, and M phases) and the endocycle (containing only the G and S phases) during trophoblast proliferation and differentiation, respectively. The cell cycle regulators p53 and Mdm2 appear to be critical for SENP2-dependent trophoblast mitosis and polyploidy. We propose that the SENP2–Mdm2–p53 pathway has a dual role in the G–S checkpoint of mitotic division and endoreduplication (Figure 11A). Although high levels of p53 induce a G1 arrest, a low level may be necessary to go through the rest of mitosis, such as through the tetraploid checkpoint. Because of the omission of M phase in endoreduplication, repression of p53 is essential to produce polyploid cells. Our findings further suggest that SENP2-dependent SUMO modification controls the subcellular localization of Mdm2 (Figure 11B). Sumoylated Mdm2, which preferentially accumulates in the nucleus, likely cannot modulate p53, whereas desumoylated Mdm2, which can move freely to the cytoplasm, is capable of p53 degradation.

This study provides evidence to support an important function of p53, as a guardian of the genome to control polyploidy. An endoreduplication deficiency was previously observed in embryos lacking cyclin E proteins [41]. In contrast to the SENP2-null deficiencies, the loss of cyclin E proteins did not affect TGC differentiation. It is conceivable that cyclin E, which functions in late G1 phase to promote S-phase entry, acts further downstream of the SENP2–Mdm2–p53 pathway. In the SENP2 mutants, we detected alterations of this regulatory pathway not only in the stem cell niche site, but also in the differentiated trophoblast layer. A recent report found that an increased number of TGCs were detected in the p53-null placentas [42], further supporting our hypothesis. SENP2 might also be involved in a crucial step of p53-dependent aneuploidy, genome instability and tumorigenesis [43]. Polyploid cells have several different fates. They can arrest in the cell cycle mediated by the tetraploidy checkpoint, which then triggers apoptosis. However, the lack of p53 allows these cells, as they escape from the arrest to undergo multipolar mitosis, to become aneuploid [44–46]. The nature of trophoblast development provides a system to elucidate the regulatory mechanism underlying polyploidy. Because of the biochemical activity of SENP2, the SENP2-null model offers a unique opportunity to further investigate the modulation of the p53–Mdm2 circuit by SUMO in normal developmental programming of polyploidy. The knowledge obtained here might be applicable to malignant transformation processes associated with polyploidy.

SENP2 is also known as Axam, which has been shown to modulate Wnt signaling by interacting with Axin, a scaffold protein involved in targeting β-catenin for degradation [14,17]. Although biochemical studies suggested that SENP2 could regulate the canonical Wnt pathway by SUMO modulation of a LEF/TCF transcription factor [31], there was no in vivo evidence to support this idea. We failed to detect alterations in Wnt–β-catenin signaling in the SENP2 mutant placentas (SC and WH, unpublished data) although this might occur in other tissues. SUMO modification of Axin has been shown to modulate its effects on JNK signaling [36]. Neither JNK, nor the related p38 and Erk1/2 factors that are important for placental function [32–35], seem to be involved in the SENP2-mediated trophoblast development (SC and WH, unpublished data). However, we identified the p53–Mdm2 pathway as a downstream target of SENP2. Our data imply that SUMO modification mediated by SENP2 is required for proper localization and function of Mdm2, which in turn controls p53 stability during trophoblast development. Not only does stimulation of p53 induce phenotypic defects resembling those of the SENP2 inactivation, but downregulation of p53 alleviates the trophoblast deficiencies caused by SENP2 deficiency. It is conceivable that Wnt or JNK/SAPK signaling regulated by SENP2 is critical for another cell type and lineage development. The generation of mouse models permitting conditional inactivation of SENP2 will aid these studies and determine its essential role in other developmental processes.

The loss of SENP2 disturbs the balance of SUMO modification. Although sumoylation of Mdm2 has been described [47], it was not clear whether this modification dictates subcellular distribution. Our data provide evidence that cellular distribution of Mdm2 is regulated by the SUMO pathway. Disruption of SENP2, leading to an accumulation of Mdm2 in a hyper-sumoylated state, induces its mislocalization. Many sumoylated proteins, including PML, preferentially accumulate in specific complexes called PML nuclear bodies [12]. Sumoylation of PML is essential not only for these nuclear bodies to form but also for other sumoylated proteins to concentrate there. Although the biological function of PML nuclear bodies remains largely elusive, subsequent recruitment of proteins can modulate transcription activity. It has been shown that sumoylation of PML directs p53 to nuclear bodies, leading to a stimulation of its transcriptional and pro-apoptotic activities [48,49]. These effects can be regulated by sumoylation of p53 [11,50,51]. Because of technical limitations and, more importantly, SUMO regulation of a number of p53 regulators (Mdm2, MdmX, and PML), the functional consequences of sumoylation have been difficult to elucidate. As SUMO modification of PML and p53 is a key determinant for maintaining genome integrity [12], our data imply that SENP2 might mediate this maintenance.

Using a mouse model with disruption of SENP2, this study suggests a novel role of SUMO modification in cell cycle progression and induction of polyploidy. Sumoylation, which dictates Mdm2 trafficking, is crucial for modulation of the p53–Mdm2 circuit. Further studies focusing on the detailed mechanistic switch of the SENP2–Mdm2–p53 pathway and its implications in other developmental and pathogenic processes promise important insights into the role of SUMO modification in mammalian development and disease.

## Materials and Methods

### Mouse strains.

Genomic DNA fragments containing the *SENP2* gene (Accession number NC_000082) were isolated by PCR and cloned into the pGEM vector. The 5′ arm contained sequences from the first coding exon to the beginning of the second coding exon, which encodes the first 49 amino acids of SENP2. The 3′ arm included parts of the fifth intron and the sixth coding exon. A β-galactosidase cDNA was fused in-frame to the second coding exon of *SENP2*. The *SENP2^lacZ^* ^/+^ mutant ES cell lines were generated by electroporation of the targeting vector into CSL3 ES cells [52]. Correct homologous recombination at the *SENP2* locus was confirmed by Southern blotting (Figure S1A). ES cell clones were injected into blastocysts to generate chimeras that were bred to obtain mice carrying the targeted allele. Mice were genotyped by PCR analysis using primers (G1: 5′-ctgttttctactgcagtggacac-3′, G3: 5′-gatacttgtagaaaggcctagtat-3′ and K1: 5′-taaccgtgcatctgccagtttga-3′) to identify the wild-type and mutant *SENP2* locus (Figure S1B). To delete the neo cassette flanked by two loxP sites, the *SENP2^lacZ/+^* strain was crossed with the Zp3-Cre strain as described [53]. PCR genotyping was performed to confirm the removal of neo and the presence of lacZ as described (Figure S1C) [52]. Care and use of experimental animals described in this work comply with guidelines and policies of the University Committee on Animal Resources at the University of Rochester.

### DNA and RNA.

The pCS2-SENP2 clone, containing the Myc-tagged *SENP2* cDNA, was generated by inserting a blunt-ended 1.7 kb Not1–Spe1 fragment into the blunt-ended Xho1–Xba1 sites of pCS2 vector [54]. The GFP-tagged Mdm2 expression vector (pGFP-Mdm2) was generated by ligation of a full length Mdm2 [55] and GFP (BD bioscience) cDNA fragments. The GFP-tagged Mdm2–SUMO expression vector was created by insertion of a SUMO-1 fragment [51] into the pGFP-Mdm2 plasmid. To generate the pBS-SENP2 clone for making the RNA probes, a 400 bp BamH1–EcoR1 fragment of the pCS2-SENP2 clone was cloned into the same restriction sites in pBS vector (Stratagene). To generate RNA probes for in situ hybridization, DNA plasmids pBS-Gcm1, pBS-Hand1, pCR4-PL-I, pCR4-Tpbpa, pBS-Ctsq, and pBS-SENP2 [19,21,23,56] were linearized and transcribed in vitro using RNA polymerases T3, T7, and SP6 (Promega). Plasmid DNA transfection was performed by Lipofectamine 2000 (Invitrogen)-mediated transfer with 4 μg pCS2-MT–SENP2, 1 μg pGFP-Mdm2, 1 μg pGFP-Mdm2–SUMO-1, or 10–100 nM p53 siRNA (Santa Cruz). Cells were plated (1.5 × 10^5^ cells in a 30 mm dish for protein extraction, 2 × 10^4^ cells in a 24-well dish for GFP analysis, and 5 × 10^5^ cells in a 60 mm dish for flow cytometry) 24 h prior to the transfection procedure. The transfected cells were harvested after 48 or 72 h for further analyses. Total RNA, isolated using Trizol (Invitrogen), was used to produce cDNA according to the manufacturer's instructions (SuperScript III, Invitrogen). The reverse transcription products were subject to PCR amplifications of the SENP2-lacZ fusion transcript using primers 5′-cagtctctacaatgctgcc-3′ and 5′-ctgtcactctgatctttgg-3′ (exons 3–5), primers 5′-gtgagctgatgagttctgg-3′ and 5′-gtcgctccaataactttcg-3′ (exons 4–6), primers 5′-ggaggagcagaatcatgg-3′ and 5′-ctcaaaatctcatctggtgg-3′ (exons 8–11) and primers 5′-cattaccagttggtctggtg-3′ and 5′-gctgcaataaacaagttccg-3′ (lacZ). The PCR reaction was performed by denaturation at 94 °C for 5 min and 30 cycles of amplification (94 °C for 30 s, 53 °C for 30 s, and 72 °C for 45 s), followed by a 7-min extension at 72 °C.

### Embryo and cell cultures.

Mouse blastocysts were recovered and cultured in DMEM medium containing 15% FBS, 100 μM β-mercaptoethanol, 100 μM non-essential amino acid, and 100 μg/ml penicillin-streptomycin, in a humidified 5% CO_2_ incubator at 37 °C. Cultured embryos were hatched and attached to dishes after 24–36 h. The differentiated trophoblasts became identifiable in a few days. For genotyping, cultured cells were incubated in 10 μl buffer containing 25 mM NaOH and 0.2 mM EDTA, pH 12 for 1 h at 95 °C, followed by the addition of 10 μl buffer containing 40 mM Tris-HCl, pH 5.0. Lysates were subject to PCR analysis. The *SENP2* wild-type allele was detected by a nested PCR assay. Primers 5′- ctgttttctactgcagtggacac-3′ and 5′-gctgcctggagtttatctactgtag-3′ were used for the first PCR reaction, performed with 35 cycles of amplification (94 °C for 30 s, 60 °C for 30 s, and 72 °C for 2 min 30 s), followed by a 7-min extension at 72 °C. Subsequently, the first PCR products were subject to a second PCR reaction using the method described for genotyping the *SENP2* wild-type mouse strain. For genotyping the SENP2 mutant culture, the same method for the *SENP2* mutant mouse strain was used.

To establish the TS cell lines [30], blastocysts were recovered in TS medium (RPMI-1640 medium containing 20% fetal bovine serum, 1 mM sodium pyruvate, 100 μM β-mercaptoethanol, 100 μg/ml penicillin–streptomycin), plus 25 ng/ml FGF4 and 1 ng/ml heparin. Briefly, each blastocyst was placed in a culture dish with mitomycin C-treated MEF feeders and cultured in a humidified 5% CO_2_ incubator at 37 °C. The blastocysts were hatched and attached to the dishes in 24–36 h. After 48 h, a small outgrowth from a blastocyst was formed and cultured in TS medium containing 25 ng/ml FGF4 and 1 ng/ml heparin. After 72–96 h, the outgrowths were ready to be disaggregated by the addition of 0.25% trypsin/EDTA and incubation for 3 min at 37 °C. The disaggregated cells were continuously cultured in TS medium with the presence of FGF4 and heparin. The TS cell colonies began to appear after days 6 to 10, and continued to be cultured until they were about 50% confluent. After expanding the cultures on the feeders for one or two passages, MEF-free TS cells were obtained and maintained in media containing 70% MEF-conditioned medium, 30% TS medium, 37.5 ng/ml FGF4, and 1.5 ng/ml heparin. To differentiate TS cells into TGC, cells were cultured in TS medium with no additions [30]. For BrdU labeling of the cultured cells, 30 μg/ml BrdU (Sigma) was added in the media for 1 h. The labeled cells were then fixed with methanol/acetone (1:1), followed by immunostaining analysis. For cell cycle analysis by flow cytometry, 8 × 10^5^ (for mitotic cell cycle) or 10^5^ (for endoreduplication cycle) TS cells were cultured in 6 cm dishes in TS media plus FGF4, heparin, and MEF-conditioned medium (undifferentiated medium) for 2 d, and TS media only (differentiated medium) for 6 d, respectively. Cells were then harvested by trypsinization and fixed in 70% ethanol at 4 °C for at least 24 h. Cells were then treated with RNase (1 mg/ml) for 30 min, followed by PI staining (20 μg/ml) for 10 min at room temperature. Samples were analyzed by an Epics Elite ESP (Coulter Electronics) set to collect 10,000 events. The percentage of cells in G0–G1, S, G2–M or with polyploidy were determined using ModFit LT software. For synchronizing cells in M phase, 3 μM nocodazole was added to the media. Nuclear and cytoplasmic fractionations of TS cells were extracted using an NE-PER extraction kit according to the manufacturer's protocol (PIERCE).

### In situ hybridization.

Paraffin sections were treated with buffer containing 0.1 M Tris-HCl and 0.1 M EDTA (pH 8.0) plus 1 μg/ml proteinase K for 30 min, and washed with the same buffer without proteinase K for 5 min at 37 °C. Samples were then incubated with buffer containing 0.2 M Tris-HCl (pH 8.0) and 0.1 M glycine for 10 min at room temp, followed by post-fixing with 4% paraformaldehyde in PBS buffer for 20 min and a 20-min wash in PBS buffer at room temperature. The sections were incubated in buffer containing 0.1 M triethanolamine (pH 8.0) for 10 min, followed by 0.25% (v/v) acetic anhydride in 0.1 M triethanolamine (pH 8.0) buffer for 10 min and by 2× SSC (1× SSC: 0.15 M sodium chloride and 15 mM sodium citrate, pH 5.5) buffer for 10 min. After dehydration through ethanol gradients and air drying for 2 h, sections were incubated with digoxygenin-labeled probes (1 μg/ml) in 5× SSC buffer containing 50% formamide, 50 μg/ml yeast tRNA and 1% SDS overnight at 70 °C. Samples were then washed three times with 5× SSC buffer for 15 min at 70 °C and 2× SSC buffer containing 50% formamide for 10 min at 45 °C before incubating with buffer containing 20 μg/ml RNase A, 5 U/ml RNase T1, 0.5 M sodium chloride, 10 mM Tris (pH 8.0) and 1 mM EDTA (pH 8.0) for 30 min at 37 °C. After washing with 2× SSC for 10 min at 37 °C and 0.1× SSC for 10 min at 45 °C, samples were incubated in MBST buffer containing 60 mM maleic acid, 0.15 M sodium chloride, and 0.1% Tween-20, pH 7.5 for 10 min and blocked with 10% goat serum in MBST for 2 h at room temperature. After incubating with anti-digoxygenin antibody (Roche) in the blocking buffer for overnight at 4 °C, sections were washed with NTMT buffer (100 mM sodium chloride, 100 mM Tris, pH 9.5, 50 mM magnesium chloride and 0.1 % Tween 20) and incubated in NTMT plus 2 mM levamisole overnight at 4 °C. To visualize the bound signals, samples were incubated with BM-purple (Roche) for 2 h to several days. The reaction was stopped by incubating in PBS buffer, followed by counterstaining with nuclear fast red.

### Histology, immunostaining and immunoblotting.

Samples were fixed, paraffin embedded, sectioned, and stained with hematoxylin/eosin for histological evaluation as described [57]. Tissue sections were subject to immunological staining with avidin:biotinylated enzyme complex as described [18,58]. Proteins were extracted from TS cells using M-PER reagent (PIERCE) with the addition of protease inhibitor cocktail (Sigma-Aldrich), 1 mM sodium molybdate, 1 mM sodium vanadate, and 10 mM N-ethylmaleimide, or SDS lysis buffer (2% SDS, 10% glycerol, and 50 mM Tris, pH 6.8). Protein extracts were subject to immunoblotting as described [54]. Bound primary antibodies were detected with horseradish peroxidase-conjugated secondary antibodies (Vector Lab), followed by ECL-mediated visualization (GE HealthCare) and autoradiography. Mouse monoclonal antibodies anti-actin (Thermo Fisher; 1:1,000), anti-BrdU (Thermo Fisher; 1:300), anti-Cdx2 (BioGenex; 1:1), anti-MDM2 (Santa Cruz; 1:100), and anti-SUMO-1 (Zymed; 1:2,000); rabbit polyclonal antibodies anti-calnexin (Stressgene; 1:2,000), anti-cyclin D1 (Neomarker; 1:100), anti-Ki67 (Neomarker; 1:400), anti-laminin (Sigma-Aldrich; 1:25), anti-Myc tag (CalBioChem; 1:400), anti-Oct4 (Santa Cruz; 1:200), anti-p53 (Santa Cruz; 1:50), and anti-p450scc (Chemicon; 1:200); and goat polyclonal antibody anti-lamin B (Santa Cruz; 1:100) were used as primary antibodies. BrdU incorporation analysis was performed by intraperitoneal injection of BrdU (250 μg/g of body weight) into pregnant females for 1 h. Placentas were recovered, fixed, embedded, sectioned, and subject to immunostaining as described [18,57].

## Supporting Information

Figure S1 Creation of Mice Carrying a SENP2-Null Allele (A–C) The targeted locus contains an in-frame insertion of lacZ into the second exon of *SENP2* and a pgk-neo gene, flanked by loxP sites, for positive selection. Diphtheria toxin (DTA) was used for negative selection. The pgk-neo was removed by Cre-mediated recombination to generate the null allele as described in Materials and Methods. Southern (A) and PCR analyses (B,C) examined the targeted and null alleles. (A) Using a 5′ external probe, the EcoRV-digested wild-type (WT, 10.3 kb) and knock-in (KI, 7.9 kb) bands were detected in the targeted ES cells by Southern blotting. Mice carrying either the targeted (B) or the null (C) allele were analyzed by PCR for the WT and KI alleles and the neo and lacZ genes as indicated.
(D) RT-PCR analyses detected the transcripts of *SENP2* and lacZ in the control (+/+) and homozygous (–/–) E10.5 embryos, respectively.
(692 KB TIF)

Figure S2 Programmed Cell Death Is Not Affected by the SENP2 Deletion Sections of the *SENP2^+/+^* (A, C, E, G) and *SENP2^–/–^* (B, D, F, H) extraembryonic structures were analyzed for terminal deoxynucleotidyl transferase-mediated dUTP–biotin nick end labeling (TUNEL) staining using fluorescent (green) or immunohistochemical (brown) assays at E7.5 (A,B) and E9.5 (C–H). No significant differences between the *SENP2^+/+^* and *SENP2^–/–^* were found at the trophoblast stem cell niches (A,B), labyrinth (C,D), spongiotrophoblast (E,F), or TGC layers (*n* = 2).
(I,J) TUNEL staining identifies cell death in the *SENP2^+/+^* (I) and *SENP2^–/–^* (J) TS cell cultures.
(K,L) TS (K) and mesenchymal (L) cell cultures induced for apoptosis with 50 μM dexamethasone for 24 h were also analyzed as positive controls. Fluorescently and immunohistochemically labeled samples were counterstained with DAPI and hematoxylin, respectively.
(M) The graph represents the average percentage of apoptotic cells in the *SENP2^+/+^* and *SENP2^–/–^* samples (*n* = 2), and the percentage of apoptotic cells in TS and mesenchymal (MSC) controls.
(3.15 MB TIF)

Figure S3 Development of the *SENP2^+/+^* and *SENP2^–/–^* TS Cell Lines The TS cell lines were derived from blastocysts isolated at E3.5. Immunostaining analyses of Oct4 (first row) and Cdx2 (second row) were performed on *SENP2^+/+^* ES (first column), *SENP2^+/+^* TS (second column), and *SENP2^–/–^* TS (third column) cells. Cells were counterstained by DAPI (third row). Scale bar, 50 μm.
(1.73 MB TIF)

## Abbreviations

E: embryonic day
ES: embryonic stem
ICM: inner cell mass
MEF: mouse embryonic fibroblast
PI: propidium iodide
PML: promyelocytic leukemia
RNAi: RNA interference
SUMO: small ubiquitin-related modifier
SENP2: SUMO-specific protease 2
TGC: trophoblast giant cells
TS: trophoblast stem
